# Validation of Reference Genes for Quantitative Real-Time PCR during Bicolor Tepal Development in Asiatic Hybrid Lilies (*Lilium* spp.)

**DOI:** 10.3389/fpls.2017.00669

**Published:** 2017-04-25

**Authors:** Leifeng Xu, Hua Xu, Yuwei Cao, Panpan Yang, Yayan Feng, Yuchao Tang, Suxia Yuan, Jun Ming

**Affiliations:** ^1^Institute of Vegetables and Flowers, Chinese Academy of Agricultural SciencesBeijing, China; ^2^College of Landscape Architecture, Nanjing Forestry UniversityNanjing, China

**Keywords:** *Lilium* spp., bicolor tepal, tepal development, quantitative real-time PCR, reference gene

## Abstract

Quantitative real-time PCR (qRT-PCR) is a reliable and high-throughput technique for gene expression studies, but its accuracy depends on the expression stability of reference genes. To date, several reliable reference gene identifications have been reported in *Lilium* spp., but none has been obtained for lily tepals at different developmental stages. In this study, ten candidate reference genes were selected and evaluated for their expression stability in *Lilium* ‘Tiny Padhye’ during the process of bicolor tepal development. The expression stability of these candidates was evaluated by three software programs (geNorm, NormFinder, and BestKeeper) and the comparative ΔCt method, and comprehensive stability rankings were generated by RefFinder. As a result, *TIP41-like family gene* (*TIP41*) and *actin* (*ACT*) were the best combination of reference genes for tepals at different developmental stages; *TIP41* and *F-box family gene* (*F-box*) for tepals under shading treatment; *ACT, actin11 (ACT11)*, and *elongation factor 1-α (EF1-α)* for different tissues; and *ACT, TIP41*, and *ACT11* for all samples. The selected optimal reference genes were further verified by analyzing the expression levels of *flavonoid 3′-hydroxylase* (*LhF3′H*) and *anthocyanidin 3-O-glucosyltransfersae (LhUFGT*) in tepals at different developmental stages. This study provides useful information for gene expression characterization in lilies under different experimental conditions, and can serve as a basis for similar research in other closely related species.

## Introduction

Lily (*Lilium* spp.) is one of the most important ornamental plants because of their various flower colors (yellows, oranges, pinks, reds, and whites) and color patterns (spots and bicolors). Among them, white–purple bicolor lily cultivars (e.g., ‘Tiny Padhye’) are attractive varieties because of their unique color pattern. However, the molecular mechanisms underlying a bicolor appearance in lilies remain largely unknown.

Gene expression analysis plays an important role in elucidating the molecular mechanisms underlying various biological processes (Bustin et al., 2005). qRT-PCR has been widely used as a powerful technique for monitoring gene expression profiles in different samples, due to its high sensitivity, accuracy, specificity, throughput capability, and cost-effectiveness (Bustin, 2002; Wong and Medrano, 2005; Nolan et al., 2006). However, the accuracy of relative quantification in qRT-PCR is always affected by many variable factors (RNA quality, reverse transcription efficiency, and amplification efficiency), which may cause inaccuracies in the gene expression data (Nolan et al., 2006). To ensure accurate results, it is necessary to use one or more stable reference genes to normalize the expression data of target genes.

Housekeeping genes, such as *ACT, glyceraldehyde 3-phosphate dehydrogenase* (*GAPDH*), *TUB, ubiquitin* (*UBQ*), and *18S ribosomal RNA* (*18S*), are commonly used as reference genes to normalize the expression profiles of target genes because of their essential roles in basic cellular processes, cell structure maintenance, and primary metabolism (Bustin, 2002). However, many studies have shown that the expression levels of these genes vary across different cultivars (Li J. et al., 2016; Qi et al., 2016), different tissues (Mallona et al., 2010; Klie and Debener, 2011; Zhang et al., 2013; Sudhakar Reddy et al., 2016), and treatments (Nicot et al., 2005; Li et al., 2014; Sinha et al., 2015). Therefore, it is necessary to validate reliable reference genes under different experimental conditions prior to gene expression studies (Gutierrez et al., 2008; Kosir et al., 2010; Xiao et al., 2016).

Several statistical tools, such as geNorm (Vandesompele et al., 2002), NormFinder (Andersen et al., 2004), and BestKeeper (Pfaffl et al., 2004), and the comparative ΔCt method (Silver et al., 2006), have been developed to identify appropriate reference genes for qRT-PCR analysis. A number of studies on the validation of reference genes using these tools have been reported in different plants under different experimental conditions (Mallona et al., 2010; Klie and Debener, 2011; Li et al., 2014; Sinha et al., 2015). Recently, the evaluation of reference genes has also been reported in roots, leaves, and bulbs of lilies at different development stages or under different stresses (Luo et al., 2014; Li et al., 2015a; Liu et al., 2016). However, to the best of our knowledge, no systematic validation of reference genes in lily tepals has yet been performed.

In this study, 10 candidate reference genes, namely, *TUB, cyclophilin* (*CYP*), *EF1-α, ACT, ACT11, F-box, GAPDH, TIP41-like family gene* (*TIP41*), *SAND family gene* (*SAND*), and *18S*, were assessed by qRT-PCR during various bicolor lily tepal developmental stages and in different tissues. To obtain the most suitable reference genes, five different statistical tools (geNorm, NormFinder, BestKeeper, the comparative ΔCt method, and RefFinder) were selected to evaluate the expression stability of these candidate genes. In addition, to validate the selected best-ranked reference genes, the expression levels of *LhF3’H* and *LhUFGT* in tepals at different developmental stages were investigated using each of the most stable reference genes or the combination of them in comparison to the least stable ones.

## Materials and Methods

### Plant Materials

**FIGURE 1:**
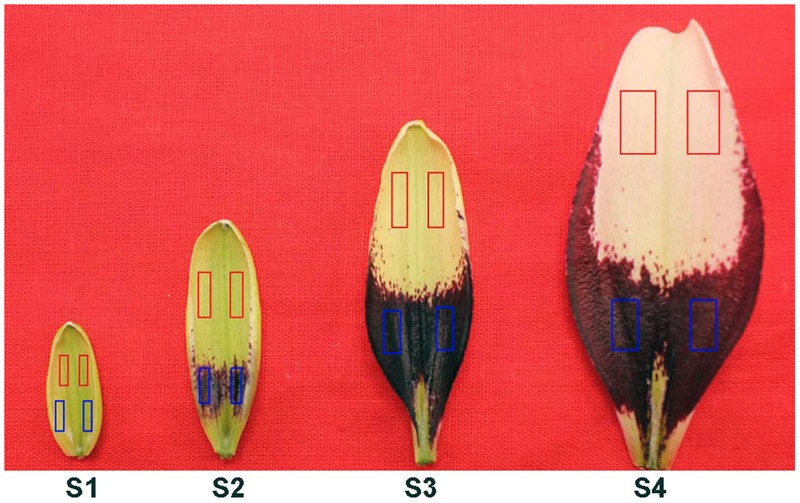
**Inner tepals of Asiatic ‘Tiny Padhye’ at four different developmental stages.** Colored boxes indicate tepal parts used for the experiments (red boxes, upper tepals; blue boxes, tepal bases).

The Asiatic lily cultivar ‘Tiny Padhye’ (a white-purple bicolor cultivar) was used in this study. Plants were grown in a greenhouse at the Chinese Academy of Agricultural Sciences (Beijing, China). No artificial light was provided. A total of 13 samples were collected. The expression stability of candidate reference genes was analyzed in the following four experimental sets. Samples from the first experimental set A represented inner tepals at four different developmental stages. Upper parts and bases of inner tepals were collected separately. Flower bud development stages (S) were as follows: stage 1 (S1; bud length of about 1.5 cm and no anthocyanin pigment is visible on tepals); stage 2 (S2; anthocyanin pigment becomes visible on tepal bases); stage 3 (S3; the day before anthesis, lower halves of tepals are fully pigmented); and stage 4 (S4; 0 day post-anthesis) (**Figure 1**). In experimental set B (shading treatment), 1.5-cm flower buds of Asiatic ‘Tiny Padhye’ were covered with silver paper, which was removed when the flowers developed to S3. Upper parts and bases of inner tepals were then immediately collected from these S3 inner tepals. Plants under natural light conditions were used as a control group. In experimental set C (plant tissues), upper parts and bases of S3 inner tepals, fresh bulb scales, stems, and leaves, were collected from untreated lily plants during flowering. The fourth experimental set D (all) was composed of all samples. The collected samples were frozen in liquid nitrogen immediately and stored at -80°C until use. Samples were obtained from 10 individual plants and three independent biological replicates were collected for each sample.

### RNA Isolation and cDNA Synthesis

Total RNA was extracted using an RNAprep Pure Plant Kit (Tiangen, Beijing, China), according to the manufacturer’s instructions. RNA integrity was confirmed by denaturing 1.0% agarose gel electrophoresis. RNA quality and quantity were determined using a NanoDrop 2000 spectrophotometer (Thermo Scientific, Waltham, MA, USA). Only the RNA samples with absorbance ratios at OD_260_/OD_280_ between 1.9 and 2.2 and OD_260_/OD_230_ greater than 2.0 were used for cDNA synthesis. First-strand cDNA synthesis was performed using a SuperScript III reverse transcription kit (Invitrogen, Carlsbad, CA, USA), according to the manufacturer’s instructions.

### Reference Gene Selection and Primer Design

Ten reference genes (commonly used in qRT-PCR) and two target genes were selected from the transcriptome of the Asiatic lily cultivar ‘Tiny Padhye’ (Xu et al., 2016). Primers for *ACT* were previously described (Xu et al., 2016). Primer pairs for genes were designed using Primer Premier 5.0 software^1^ with the following parameters: melting temperatures (T_m_) of 55–65°C, primer lengths of 17–25 bp, and amplicon lengths of 100–300 bp (**Table 1**). Primer specificity was assessed using gel electrophoresis and melting-curve analyses.

**Table 1:** Primers used for quantitative real-time PCR analyses.

Unigene ID	Gene symbol	Gene name	Primer sequence (5′-3′)	Product length (bp)	*R*^2^	E (%)
>c92708_g1	*TUB*	*Tubulin*	CGAGCACGGCATAGACAA	285	0.996	93.965
			CGCACAACATCAAGCACC			
>c56451_g1	*CYP*	*Cyclophilin*	CCCGAATACGAATGGCTCA	164	0.997	91.636
			CAATCACCACCTTGGCAGAA			
>c105281_g1	*EF1-α*	*Elongation factor 1-α*	AGCCAAGGTTACCAAGTCT	263	0.996	96.862
			TCAGCAGTACCAGCATCAC			
>c112398_g1	*ACT*	*Actin*	GCACCTGAAGAGCACCCT	145	0.998	91.464
			TGGCGTAAAGCGACAAAA			
>c122623_g8	*ACT11*	*Actin11*	CACTGCTGAGCGGGAAAT	190	0.998	89.590
			TGATGGCTGGAAGAGGAC			
>c96615_g1	*F-box*	*F-box family*	TCGGCACAAGCAAAGTCA	252	0.997	96.928
			ACTGGGAGGTGTTAGGGGAC			
>c117539_g2	*GAPDH*	*Glyceraldehyde 3-phosphate dehydrogenase*	GCTGCAAGTTTCAACATTGTTCC	266	0.997	93.263
			ATCATAAGTAGCCGCCTTCTCA			
>c109800_g1	*TIP41*	*TIP41-like family*	ATCAGGGTAGGGTGGATTGG	183	0.998	89.942
			GGTTTGGCTTTTGGGTCGTT			
>c117408_g1	*SAND*	*SAND family*	GAGAATGGTGAAGACCGTGTC	236	0.994	95.515
			TCTGTTCCTCCCAGCAATG			
>c9661152_g1	*18S*	*18S ribosomal RNA*	TAATTCTCCGTCACCCGTCAC	178	0.998	102.250
			CAATACCGGGCGCTTTAGTGT			
>c113932_g1	*LhF3’H*	*Flavonoid 3′-hydroxylase*	ACTGAAATCAAGGCGTTGTTAC	229	0.945	91.812
			ACGGATGGAGTCGGAAAGT			
>c117809_g1	*LhUFGT*	*Anthocyanidin 3-O-glucosyltransfersae*	CCCACAATGCGTCACAAA	117	0.995	96.409
			CAGTTGCCTCAAGGGTTTT			

### qRT-PCR Analysis

qRT-PCR analysis was performed using SYBR^®^ Premix Ex Taq^TM^ II (Tli RNaseH Plus) (Takara, Dalian, China) and a Bio-Rad CFX96 system (Bio-Rad, Hercules, CA, USA) with the following reaction conditions: initial denaturation at 95°C for 30 s, 40 cycles of 95°C for 5 s and 60°C for 30 s, and a melting-curve program (65–95°C with a temperature increment of 0.5°C every 5 s). The melting curve was created to identify the amplicon specificity. All reactions were performed with three biological replicates and three technical replicates. The primer efficiency of each gene and regression coefficient (*R*^2^) were evaluated using a standard curve generated from a fivefold dilution series of cDNA (1/5, 1/25, 1/125, 1/625). A no-template control and a reverse transcription negative control were included to monitor the potential reagents and genomic DNA contamination, respectively.

### Gene Expression Stability Analysis

To show the variation in the expression of each gene, boxplots of quantitative cycle (Cq) values for the 10 candidate reference genes were drawn using the boxplot R package. Expression stability of these 10 reference genes under different experimental conditions was analyzed using geNorm (Vandesompele et al., 2002), NormFinder (Andersen et al., 2004), BestKeeper (Pfaffl et al., 2004), and the comparative ΔCt method (Silver et al., 2006). All of these software tools were run in accordance with their manuals. Furthermore, comprehensive stability rankings were generated using the web-based tool RefFinder^2^. It should be noted that the data of each biological replicate were analyzed separately.

### Validation of Identified Reference Genes

Two lily anthocyanin biosynthesis-related genes, *LhF3′H* and *LhUFGT*, were selected as target genes to validate the reliability of the identified reference genes. Their gene expression profiles in the upper parts and bases of inner tepals at different developmental stages were normalized using the most stable candidate reference gene(s) as well as the least stable reference gene(s). Sample collections and experiments were performed as described above. The average Cq value was calculated from three biological replicates and three technical replicates and used for relative expression analyses. Relative gene expression levels of *LhF3’H* and *LhUFGT* were calculated using the 2^-ΔΔCq^ method (Livak and Schmittgen, 2001).

## Results

### Assessment of Primer Specificity and PCR Amplification Efficiency

**FIGURE 2:**
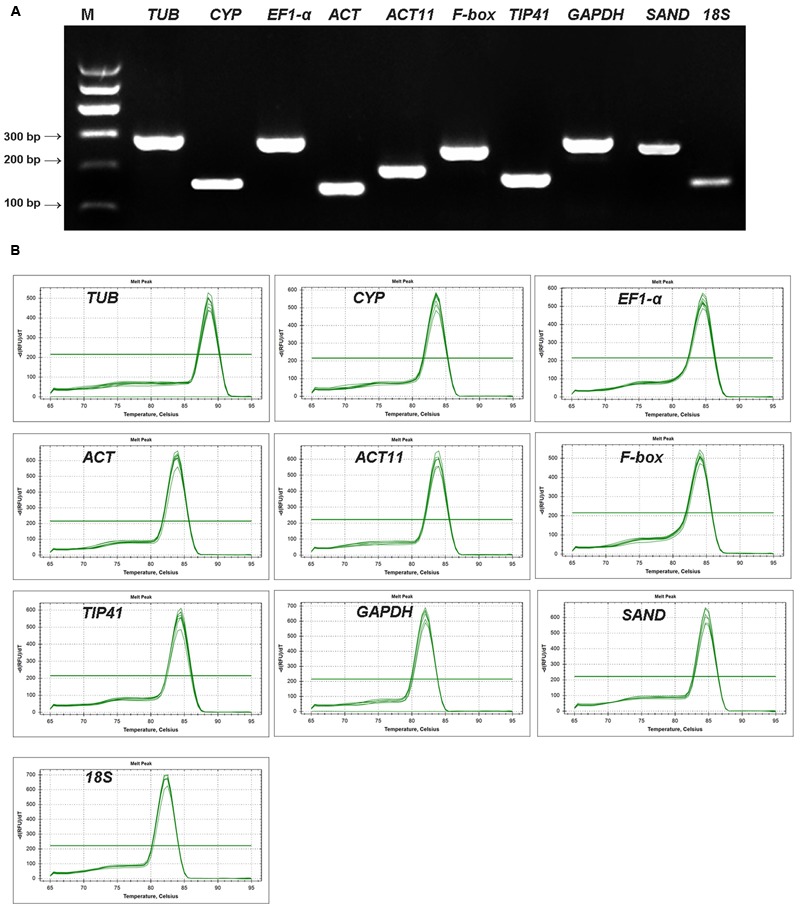
**Amplification specificity of primers for qRT-PCR amplification. (A)** Amplified fragments of ten candidate genes on 2% agarose gel. **(B)** Melting curves of 10 candidate reference genes.

A total of 10 candidate reference genes were selected from the transcriptome of the Asiatic lily cultivar ‘Tiny Padhye’ for qRT-PCR analysis. The presence of a single PCR product with the expected size and single peak in the melting curve analysis for each gene confirmed the specific amplification of each gene (**Figure 2**). The amplification efficiency (E) of all PCR reactions ranged from 89.59% for *ACT11* to 102.25% for *18S* (**Table 1**), suggesting that these genes are suitable for further gene expression analysis. Meanwhile, the standard curves showed good linear relationships, with correlation coefficients (*R*^2^) above 0.99 (**Table 1**).

### Cq Values of Candidate Reference Genes

**FIGURE 3:**
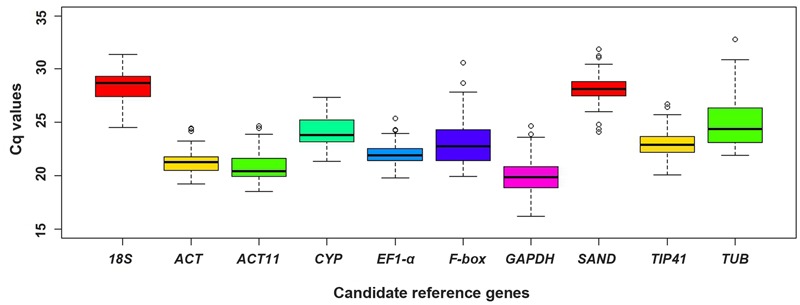
**Distribution of Cq values of candidate reference genes in all samples.** Boxplots show the 25th and 75th percentiles, mean, and outliers.

To assess the expression stability of the reference genes in different samples, the transcript abundances of 10 candidate reference genes were presented as their Cq values, which varied from 16.22 (*GAPDH*) to 32.76 (*TUB*), while the mean Cq values varied from 20.02 (*GAPDH*) to 28.39 (*18S*) (**Figure 3**).

### Analysis of Reference Gene Stability Using Five Bioinformatic Programs

The orders of candidate gene stability ranking under different experimental conditions were determined separately using geNorm, NormFinder, BestKeeper, the comparative ΔCt method, and RefFinder.

**FIGURE 4:**
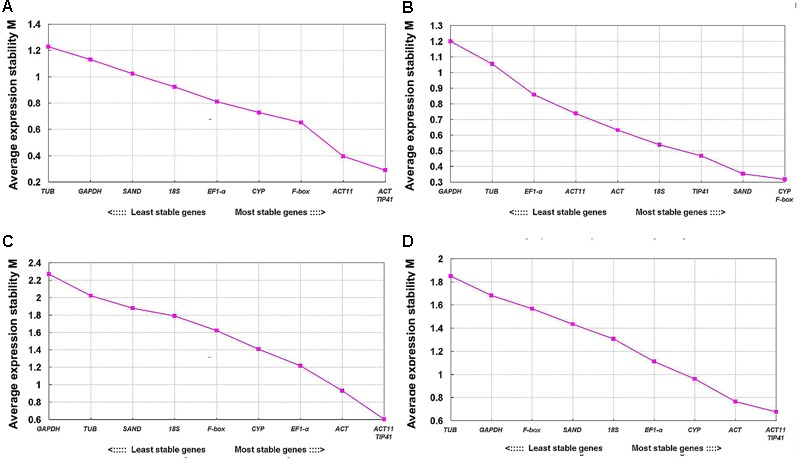
**Expression stability values (*M*) of 10 candidate reference genes calculated by geNorm. (A)** Upper parts and bases of inner tepals at different developmental stages, **(B)** tepals under shading treatment, **(C)** different tissues, and **(D)** all samples.

The geNorm program is used to rank the stability of expression of tested genes by calculating their expression stability values (*M*) based on the average pairwise expression ratio (Vandesompele et al., 2002). Candidate reference genes with lower average expression stability values (*M*) are considered to be more stably expressed reference genes (Vandesompele et al., 2002). The stability values (*M*) of tested genes evaluated by geNorm are shown in **Figure 4**. *TIP41* and *ACT* were ranked as the two most stable genes for tepals at different developmental stages, and *ACT11* and *TIP41* for different tissues and all samples, while *TUB* and *GAPDH* were the two least stable genes. For tepals under shading treatment, *CYP* and *F-box* were the two most stable genes with the lowest *M*-value.

**FIGURE 5:**
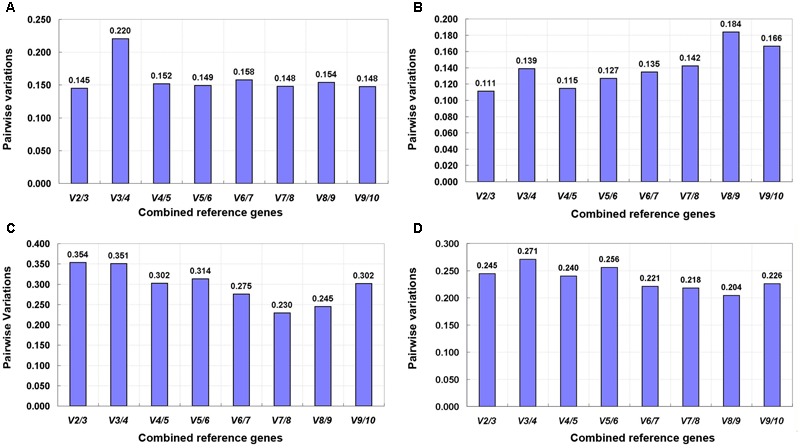
**Pairwise variation (V) of 10 candidate reference genes calculated by geNorm. (A)** Upper parts and bases of inner tepals at different developmental stages, **(B)** tepals under shading treatment, **(C)** different tissues, and **(D)** all samples.

The pairwise variation (V_n_/V_n + 1_) value calculated by the geNorm algorithm determines the optimal number of reference genes for gene expression normalization. A value of V_n_/V_n + 1_ < 0.15 suggests that the optimal number of reference genes equal to a value of n is sufficient (Vandesompele et al., 2002). In this study, the value of V_2_/V_3_ was below 0.15 for tepals at different developmental stages and under shading treatment (**Figures 5A,B**), indicating that two reference genes would be sufficient for gene normalization under these experimental conditions. However, when different tissues and total samples were considered, all V_n_/V_n + 1_ values were still above 0.15 (**Figures 5C,D**), and thus the cut-off value of 0.15 was somewhat strict in these cases. Many studies have shown that the use of 0.15 as a cut-off value is just a recommendation, and whether 0.15 is actually used is dependent on the data (Silveira et al., 2009; Fernandez et al., 2011; Lu et al., 2013; De Lima et al., 2016). Generally, a reliable result could be obtained by using three reference genes in the majority of experiments (Toegel et al., 2007; Maroufi et al., 2010; Wang et al., 2014; Wang and Lu, 2015; Li M.Y. et al., 2016). Considering this, we opted to use three reference genes in these experimental conditions.

NormFinder ranks the stability of expression of candidate reference genes by calculating the average pairwise variation in a gene relative to that of other candidate genes (Andersen et al., 2004). Genes with the lowest expression stability value are characterized as the most stable reference genes (Andersen et al., 2004). The stability values of tested genes evaluated by NormFinder are listed in **Table 2**. For different tissues, the most stable gene was *ACT*, followed by *ACT11* and *EF1-α*. For tepals under shading treatment, *TIP41* and *F-box* were the two most stable genes. For tepals at different developmental stages, the three most stable genes were *TIP41, ACT*, and *ACT11*. Finally, *ACT* was the most stable reference gene while *TUB* was the least stable one for all samples.

**Table 2:** Expression stability of candidate reference genes.

Method	1	2	3	4	5	6	7	8	9	10
**Ranking order of candidate reference genes in all samples (Better–Good–Average)**					
ΔCt	*ACT*	*TIP41*	*ACT11*	*CYP*	*EF1-α*	*18S*	*F-box*	*SAND*	*GAPDH*	*TUB*
BestKeeper	*EF1-α*	*ACT*	*SAND*	*TIP41*	*CYP*	*ACT11*	*18S*	*GAPDH*	*F-box*	*TUB*
NormFinder	*ACT*	*TIP41*	*ACT11*	*CYP*	*EF1-α*	*18S*	*F-box*	*SAND*	*GAPDH*	*TUB*
geNorm	*ACT11 | TIP41*		*ACT*	*CYP*	*EF1-α*	*18S*	*SAND*	*F-box*	*GAPDH*	*TUB*
RefFinder	*ACT*	*TIP41*	*ACT11*	*EF1-α*	*CYP*	*SAND*	*18S*	*F-box*	*GAPDH*	*TUB*
**Ranking order of candidate reference genes in different tissues (Better–Good–Average)**					
ΔCt	*ACT*	*ACT11*	*TIP41*	*EF1-α*	*CYP*	*18S*	*F-box*	*SAND*	*TUB*	*GAPDH*
BestKeeper	*EF1-α*	*CYP*	*SAND*	*ACT*	*18S*	*TIP41*	*ACT11*	*F-box*	*TUB*	*GAPDH*
NormFinder	*ACT*	*ACT11*	*EF1-α*	*TIP41*	*CYP*	*18S*	*F-box*	*SAND*	*TUB*	*GAPDH*
geNorm	*ACT11 | TIP41*		*ACT*	*EF1-α*	*CYP*	*F-box*	*18S*	*SAND*	*TUB*	*GAPDH*
RefFinder	*ACT*	*ACT11*	*EF1-α*	*TIP41*	*CYP*	*18S*	*SAND*	*F-box*	*TUB*	*GAPDH*
**Ranking order of candidate reference genes in tepals under shading treatment (Better–Good–Average)**					
ΔCt	*TIP41*	*SAND*	*F-box*	*CYP*	*ACT*	*18S*	*ACT11*	*EF1-α*	*TUB*	*GAPDH*
BestKeeper	*ACT*	*ACT11*	*EF1-α*	*TIP41*	*TUB*	*F-box*	*SAND*	*18S*	*CYP*	*GAPDH*
NormFinder	*TIP41*	*F-box*	*SAND*	*CYP*	*18S*	*ACT*	*ACT11*	*EF1-α*	*TUB*	*GAPDH*
geNorm	*CYP | F-box*		*SAND*	*TIP41*	*18S*	*ACT*	*ACT11*	*EF1-α*	*TUB*	*GAPDH*
RefFinder	*TIP41*	*F-box*	*SAND*	*CYP*	*ACT*	*ACT11*	*18S*	*EF1-α*	*TUB*	*GAPDH*
**Ranking order of candidate reference genes in tepals at different developmental stages (Better–Good–Average)**					
ΔCt	*TIP41*	*ACT*	*ACT11*	*F-box*	*CYP*	*EF1-α*	*18S*	*GAPDH*	*SAND*	*TUB*
BestKeeper	*ACT*	*TIP41*	*EF1-α*	*ACT11*	*SAND*	*18S*	*F-box*	*CYP*	*GAPDH*	*TUB*
NormFinder	*TIP41*	*ACT*	*ACT11*	*F-box*	*CYP*	*EF1-α*	*18S*	*GAPDH*	*SAND*	*TUB*
geNorm	*ACT | TIP41*		*ACT11*	*F-box*	*CYP*	*EF1-α*	*18S*	*SAND*	*GAPDH*	*TUB*
RefFinder	*TIP41*	*ACT*	*ACT11*	*F-box*	*EF1-α*	*CYP*	*18S*	*SAND*	*GAPDH*	*TUB*

BestKeeper is another software tool for determining the expression stability of reference genes by calculating the coefficient of variation (CV) and the standard deviation (SD) of Cq values (Pfaffl et al., 2004). The most stable reference genes are identified as those with the lowest CV and SD (Pfaffl et al., 2004). In this study, *EF1-α* and *CYP* were identified as the most stable genes for different tissues, *ACT* and *ACT11* for tepals under shading treatment, *ACT* and *TIP41* for tepals at different developmental stages, and *EF1-α* and *ACT* for all samples (**Table 2**).

The comparative ΔCt method identifies potential reference genes by comparing the relative expression of gene pairs within each sample (Silver et al., 2006). The ranking orders of the ten tested genes generated by the ΔCt method are shown in **Table 2**. *ACT, TIP41*, and *ACT11* were the three most stable genes for tepals at different flower developmental stages, different tissues, and all samples. For tepals under shading treatment, the most stable gene was *TIP41*, followed by *SAND* and *F-box* (**Table 2**).

The ranking orders of the ten tested genes generated by geNorm, NormFinder, BestKeeper, and the comparative ΔCt method showed some differences (**Table 2**). To provide a comprehensive evaluation of candidate reference genes, further analysis was thus carried out using the web-based comprehensive tool RefFinder, which integrates geNorm, Normfinder, BestKeeper, and the comparative ΔCt method. The comprehensive ranking orders recommended by RefFinder are shown in **Table 2**. For all samples and tepals at different developmental stages, *TIP41, ACT*, and *ACT11* were ranked as the top three most stable genes. *TIP41* and *F-box* were found to be the most stable genes for tepals under shading treatment, while *ACT, ACT11*, and *EF1-α* were the most stable genes for different tissues.

The best combination of reference genes in different experimental sets was determined based on the optimal number of reference genes calculated by geNorm and the ranking list obtained using RefFinder. Specifically, *TIP41* and *ACT* were found to be the best combination of reference genes for tepals at different developmental stages, *TIP41* and *F-box* for tepals under shading treatment, *ACT, ACT11*, and *EF1-α* for different tissues, and *ACT, TIP41*, and *ACT11* for all samples.

### Validation of the Identified Reference Genes

**FIGURE 6:**
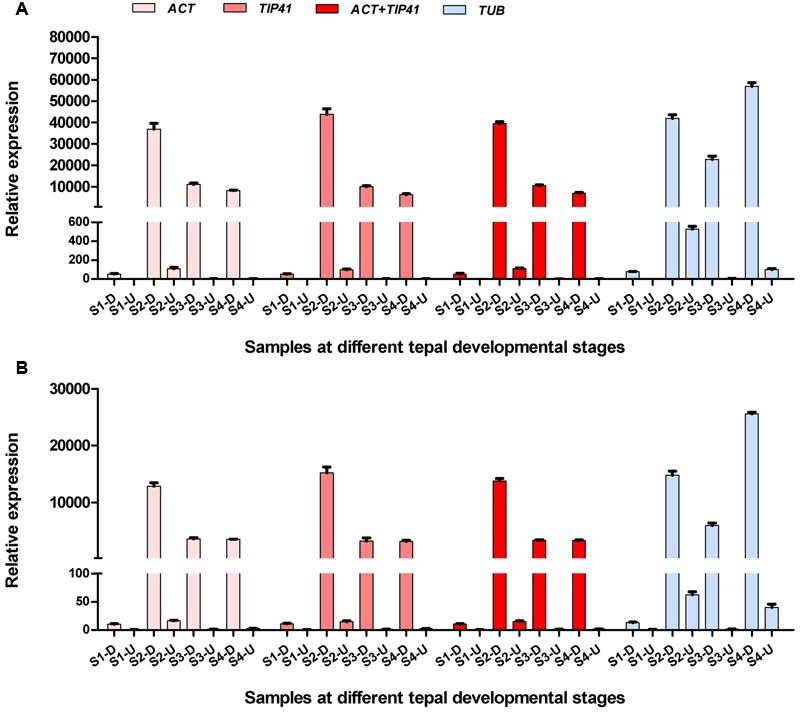
**Relative expression patterns of *LhF3′H* (A)** and *LhUFGT* **(B)** in upper parts and bases of inner tepals at different developmental stages. S1-D, S2-D, S3-D, and S4-D mean inner tepal bases at S1, S2, S3, and S4, respectively. S1-U, S2-U, S3-U, and S4-U mean upper parts of inner tepals at S1, S2, S3, and S4, respectively. Error bars for qRT-PCR show the standard deviation of three replicates.

To validate the selected candidate reference genes, the expression levels of two anthocyanin biosynthesis-related genes (*LhF3′H* and *LhUFGT*) were investigated using different reference genes in inner tepals at different developmental stages. Each of the two most stable reference genes (*ACT* and *TIP411*), combination of stable genes (*ACT* + *TIP41*), and the least stable reference gene (*TUB*) were used as internal controls. When using *ACT* alone, *TIP41* alone, and the combination of *ACT* + *TIP41* for normalization, *LhF3′H* and *LhUFGT* showed the highest expression in tepal bases at S2 and an extremely low level of expression in upper tepals at all four stages (**Figure 6**). However, when the least stable gene *TUB* was used for normalization, the expression patterns were completely different. Namely, *LhF3′H* and *LhUFGT* had the highest expression levels in tepal bases at S4 (**Figure 6**).

## Discussion

qRT-PCR has become an important technique for the analysis of gene expression because of its sensitivity, accuracy, and high throughput (Bustin, 2002). However, it is necessary to validate reliable reference genes under different experimental conditions prior to qRT-PCR analysis. In this study, the expression stability of 10 candidate reference genes was analyzed in Asiatic ‘Tiny Padhye’ under different experimental conditions. This is the first systematic study on the selection of reliable stable reference genes for qRT-PCR analysis in lily tepals at different developmental stages.

Three software programs (geNorm, NormFinder, and BestKeeper) and the comparative ΔCt method were used to evaluate the expression stability of candidate reference genes in our analysis. The ranking orders of the 10 tested genes generated by different algorithms showed some substantial discrepancies (**Table 2**). For instance, in tepals under shading treatment, *CYP* and *F-box* were the best reference genes identified by geNorm (**Table 2** and **Figure 4**), while *ACT* was identified as the most stable reference gene by BestKeeper (**Table 2**), and *TIP41* was the best reference gene predicted by NormFinder and the comparative ΔCt method (**Table 2**). Differences in rankings among these programs have also been reported in other studies (Tong et al., 2009; Delporte et al., 2015; Mangeot-Peter et al., 2016; Sudhakar Reddy et al., 2016), which is likely the result of the different statistical models that they employ. In this study, to provide a comprehensive evaluation of candidate reference genes, the web-based tool RefFinder was used to generate comprehensive stability rankings, and then the best combinations of reference genes in different subsets were determined based on the optimal number of reference genes calculated by geNorm (**Figure 5**) and the ranking list obtained using RefFinder (**Table 2**).

In this study, the best combinations of reference genes in different experimental conditions are different. *TIP41* and *ACT* were the best combination of reference genes for tepals at different developmental stages; *TIP41* and *F-box* for tepals under shading treatment; *ACT, ACT11*, and *EF1-α* for different tissues; and *ACT, TIP41*, and *ACT11* for all samples. Additionally, *GAPDH* shows stable expression in different tissues of *Lilium brownii* (Luo et al., 2014) and *Lilium davidii* var. Unicolor (Li et al., 2015a). Nevertheless, in our study, it was found to be the least stable reference gene in different tissues of Asiatic ‘Tiny Padhye,’ while *ACT* was the most stable reference gene. These confirm the importance of validating reliable reference genes prior to qRT-PCR analysis under particular experimental conditions.

To validate the suitability of the identified reference genes, the expression patterns of *LhF3′H* and *LhUFGT* were investigated in tepals at different developmental stages using different reference genes. The data once again demonstrated that reference genes play a key role in normalizing the data from qRT-PCR, and the use of inappropriate reference genes may lead to inaccurate results.

There are some limitations in this study that should be mentioned. To avoid the effect of gDNA contamination, intron-spanning primer pairs are regarded as ideal primers for qRT-PCR analysis (Kosir et al., 2010; Eildermann et al., 2012; Carvalho et al., 2015). Unfortunately, since genomic resources for *Lilium* spp. are still scarce, it is difficult to design intron-spanning primer pairs for the candidate reference genes. However, in this study, a reverse transcription negative control was included to monitor potential genomic DNA contamination. Furthermore, selected reference genes here were limited to 10 traditional housekeeping genes. An increasing number of studies have identified other novel reference genes, such as *clathrin adaptor complexes medium subunit family protein* (*GhMZA*) (Artico et al., 2010), *clathrin adaptor complexes* (*CAC*) (Xiao et al., 2014), *calmodulin-like domain protein kinase* (*CDPK*) (Li et al., 2015b), *histone H3* (*HIS*) (Zhuang et al., 2015), and *ribosomal protein L17* (*RPL17*) (Huang et al., 2016), which can also be evaluated for their expression stability in *Lilium* spp. in the future.

## Author Contributions

JM designed the research. LX, HX, YC, PY, YT, SY, and YF conducted the experiments. LX analyzed the data and wrote the manuscript.

## Conflict of Interest Statement

The authors declare that the research was conducted in the absence of any commercial or financial relationships that could be construed as a potential conflict of interest.

## Footnotes

1 http://www.premierbiosoft.com/

2 http://www.leonxie.com/referencegene.php
